# Rheumatologische Weiterbildungsstellen in Deutschland

**DOI:** 10.1007/s00393-022-01284-4

**Published:** 2022-10-20

**Authors:** Alexander Pfeil, Martin Krusche, Fabian Proft, Diana Vossen, Jürgen Braun, Xenofon Baraliakos, Michael N. Berliner, Gernot Keyßer, Andreas Krause, Hanns-Martin Lorenz, Bernhard Manger, Florian Schuch, Christof Specker, Jürgen Wollenhaupt, Anna Voormann, Martin Fleck

**Affiliations:** 1 Kommission Fort- und Weiterbildung der Deutschen Gesellschaft für Rheumatologie, Berlin, Deutschland; 2 Klinik für Innere Medizin III, Funktionsbereich Rheumatologie und Osteologie, Universitätsklinikum Jena, Am Klinikum 1, 07747 Jena, Deutschland; 3 Sektion für Rheumatologie und Entzündliche Systemerkrankungen, Universitätsklinikum Hamburg-Eppendorf (UKE), Hamburg, Deutschland; 4 Abteilung für Rheumatologie, Medizinische Klinik für Gastroenterologie, Infektiologie und Rheumatologie, Campus Benjamin Franklin, Charité Universitätsmedizin, Berlin, Deutschland; 5 Rheinisches Rheumazentrum Meerbusch-Lank, St. Elisabeth Hospital, Meerbusch, Deutschland; 6 Rheumazentrum Ruhrgebiet, Ruhr-Universität Bochum, Herne, Deutschland; 7 Rheumatologie und Geriatrie, Helios Klinikum Berlin-Buch, Berlin, Deutschland; 8 Department für Innere Medizin, Klinik für Innere Medizin II, Universitätsklinikum Halle, Halle (Saale), Deutschland; 9 Klinik für Innere Medizin, Abteilung Rheumatologie, klinische Immunologie und Osteologie, Immanuel Krankenhaus Berlin, Berlin, Deutschland; 10 Sektion Rheumatologie, Medizinische Klinik V, Universitätsklinikum Heidelberg, Heidelberg, Deutschland; 11 Medizinische Klinik 3, Rheumatologie und Immunologie, Universitätsklinikum Erlangen, Friedrich-Alexander Universität Erlangen-Nürnberg, Erlangen, Deutschland; 12 Internistische Praxisgemeinschaft Rheumatologie – Nephrologie, Erlangen, Deutschland; 13 Klinik für Rheumatologie und Klinische Immunologie, Evangelisches Krankenhaus Kliniken Essen-Mitte, Essen, Deutschland; 14 Immunologikum Hamburg, Hamburg, Deutschland; 15 Deutsche Gesellschaft für Rheumatologie, Berlin, Deutschland; 16 Klinik und Poliklinik für Innere Medizin I, Universitätsklinikum Regensburg, Regenburg, Deutschland; 17 Asklepios Klinikum Bad Abbach, Klinik und Poliklinik für Rheumatologie/Klinische Immunologie, Bad Abbach, Deutschland

**Keywords:** Weiterbildung, Fachgebiet Innere Medizin und Rheumatologie, Weiterbildungsorte, Kliniken, Niederlassung, Advanced training, Speciality of internal medicine and rheumatology, Training location, Clinical sector, Outpatient sector

## Abstract

**Hintergrund:**

In den nächsten Jahren gehen viele Haus- bzw. Fachärzt:innen in den Ruhestand. Wie in anderen Disziplinen stellt sich in der Rheumatologie die Frage, ob ausreichend Weiterbildungsstellen zur Verfügung stehen, um das Versorgungsangebot bedarfsgerecht aufrechterhalten bzw. ausweiten zu können. Daher hat die Deutsche Gesellschaft für Rheumatologie (DGRh) ihre Kommission Fort- und Weiterbildung beauftragt, die aktuell zur Verfügung stehenden Weiterbildungsmöglichkeiten in Deutschland zu überprüfen. Ziel dieser Arbeit ist die Erfassung der Weiterbildungskapazität zur Fachärztin bzw. zum Facharzt für Innere Medizin und Rheumatologie.

**Methodik:**

Im Rahmen dieser Studie erfolgte die Erhebung der Weiterbildungsbefugten, deren Tätigkeitsort und die Dauer von deren Weiterbildungsbefugnis über die Homepages der 17 Landesärztekammern. Basierend auf diesen Daten erfolgte dann eine deutschlandweite Umfrage zu den Weiterbildungsstellen.

**Ergebnisse:**

Die Weiterbildung zum/zur Facharzt/Fachärztin für Rheumatologie erfolgte in Deutschland im Jahr 2021 an 229 Weiterbildungsorten. Dabei standen von 187 Weiterbildungsorten nähere Daten für eine Analyse zur Verfügung (81,7 %). Die Weiterbildungsorte verteilten sich dabei auf Kliniken (52,4 %) und Niederlassungen (47,6 %), wobei der Großteil (81,8 %) der insgesamt 478,4 Weiterbildungsstellen (Klinik: 391,4 und Niederlassung: 87) im Krankenhausbereich lag. Insgesamt waren zum Erhebungszeitpunkt 17,2 % aller Weiterbildungsstellen (Klinik: 11,4 % und Niederlassung: 43,1 %) nicht besetzt.

**Diskussion:**

Die Studie zeigt, dass die meisten Weiterbildungsstellen in klinischen Einrichtungen vorhanden sind. Demgegenüber gibt es im niedergelassenen Bereich vergleichsweise wenige Weiterbildungsstellen, die zudem zur Hälfte nicht besetzt sind. Für eine optimale Nutzung bereits bestehender Weiterbildungskapazitäten müssen sektorübergreifende Weiterbildungskonzepte entwickelt und v. a. muss auch eine eigenständige Vergütung des Weiterbildungsaufwandes etabliert werden. In diesem Kontext muss eine gute rheumatologische Versorgung in ganz Deutschland dauerhaft gewährleistet sein, um den betroffenen ca. 2 Mio. Patienten mit entzündlich rheumatischen Erkrankung gerecht werden zu können.

## Abstract (en)

**Background:**

In the next few years many general practitioners and specialists will retire. As in other disciplines the question arises in rheumatology whether sufficient training positions are available to maintain or expand the supply of care according to demand. Therefore, the German Society of Rheumatology (DGRh) has assigned its committee for education and training to review the currently available training opportunities in Germany. The aim of this work is the quantitative survey of the training capacity to become a specialist in internal medicine and rheumatology.

**Methods:**

Within the framework of this study, a survey was conducted via the homepages of the 17 state medical associations to determine the postgraduate medical officers, their place of work and the duration of their postgraduate training capabilities. Based on the data, a nationwide survey of training positions was conducted.

**Results:**

Specialized rheumatology training is established at 229 training centers in Germany, whereby data from 187 training sites were available for analysis. The training locations are distributed as followed: 52.4% clinical sector and 47.6% outpatient sector. In total, 478.4 training positions are available in Germany (clinical sector: 391.4 and outpatient sector: 87) and 17.2% of the positions (clinical sector: 11.4% and outpatient sector: 43.1%) are not occupied.

**Conclusion:**

Based on this study, it can be shown that most of the continuing education positions are available in the clinical sector. In contrast, half of the training positions in the outpatient area are not filled. In order to improve the training situation, it is essential to integrate outpatient colleagues into the training program. This presupposes that further training is supported or financed by the healthcare system. In this context, optimal rheumatological care must be permanently guaranteed throughout Germany in order to provide sufficient care for the approximately 2 million patients with inflammatory rheumatic diseases.

Der Bedarf an Fachärztinnen und Fachärzten für Innere Medizin und Rheumatologie ist groß und wird angesichts des Alterungsprozesses der Gesellschaft auch noch weiter steigen. Der bevorstehende Generationenwechsel und auch neue Arbeitszeitmodelle stellen nicht nur unser Fachgebiet vor große Herausforderungen [7, 11, 16].

Dem Memorandum der Deutschen Gesellschaft für Rheumatologie (DGRh) aus dem Jahr 2017 entsprechend ist der Bedarf an Fachärztinnen und Fachärzten für Innere Medizin und Rheumatologie in Deutschland nicht ausreichend gedeckt. Basierend auf der errechneten Bedarfszahl von 1 Rheumatolog:in auf 50.000 Einwohner fehlen im ambulanten und stationären sowie auch im rehabilitativen Bereich Rheumatolog:innen [16]. Entsprechend der Arbeit von Fiehn et al. war 2020 ca. ein Drittel der niedergelassenen Rheumatolog:innen 60 Jahre oder älter [3]. Des Weiteren konnte in einer Umfrage von Keyßer et al. im Jahr 2019 gezeigt werden, dass in den nächsten 15 Jahren ca. 50 % der Facharztpositionen aus Altersgründen neu besetzt werden müssen [7]. Demgegenüber stehen im Jahr 2020 63 Facharztanerkennungen [6]. Darüber hinaus ist durch den demografischen Wandel eine Zunahme der Prävalenz von entzündlich rheumatischen Krankheitsbildern zu erwarten [1, 15].

Unter Berücksichtigung des bereits bestehenden Facharztmangels, des bevorstehenden Generationswechsels sowie der zu erwartenden Zunahme entzündlich rheumatischer Erkrankungen besteht die dringende Notwendigkeit der Aus- und Weiterbildung neuer Fachärztinnen und Fachärzte für Innere Medizin und Rheumatologie [12].

Aktuelle Daten bezüglich der vorhandenen Weiterbildungsorte und Weiterbildungsstellen lagen in Deutschland bis dato nicht vor. Aus diesem Grund erfolgte im Rahmen der Studie die Erhebung der Weiterbildungsorte und Weiterbildungsstellen für Rheumatolog:innen in Deutschland.

## Methoden

Die Studie gliedert sich in eine Online-Recherche der Webseiten der Landesärztekammern bezüglich der Weiterbildungsbefugten für Innere Medizin und Rheumatologie sowie folgend in eine Befragung der Weiterbildungsbefugten für die entsprechende Facharztbezeichnung auf.

### Online-Recherche der Webseiten der Landesärztekammern

Anhand einer Online-Recherche der Webseiten der Landesärztekammern wurden die Weiterbildungsbefugten für Innere Medizin und Rheumatologie, die dazugehörigen Klinik- bzw. Praxisadresse, Weiterbildungsdauer sowie die Weiterbildungsform (Einzelweiterbildung vs. Verbundweiterbildung) ermittelt. Insgesamt konnten 236 Weiterbildungsorte ermittelt werden.

### Umfrage der Weiterbildungsbefugten

Basierend auf den Daten der Online-Recherche wurden die Weiterbildungsbefugten durch die Geschäftsstelle der DGRh angeschrieben. Dem Anschreiben lag ein Fragebogen zu folgenden Fragestellungen zum 01.01.2021 bei:

1. Anzahl der Weiterbildungsstellen
2. Anzahl der besetzten Weiterbildungsstellen
3. Anzahl der freien Weiterbildungsstellen
4. Wie viele Stellen sind in Teilzeit besetzt (die Teilzeitstellen wurden wie folgt definiert: 80 %, 75 %, 60 %, 50 % und 40 %)?

Alternativ konnte der Fragebogen über eine Online-Umfrage (SurveyMonkey®) beantwortet werden. Wurde kein Eingang einer Antwort in der Geschäftsstelle der DGRh bis zum 31.05.2021 registriert, erfolgte eine erneute Kontaktierung der Weiterbildungsbefugten mittels E‑Mail, Telefax oder eines Telefoninterviews.

**Figure 1:**
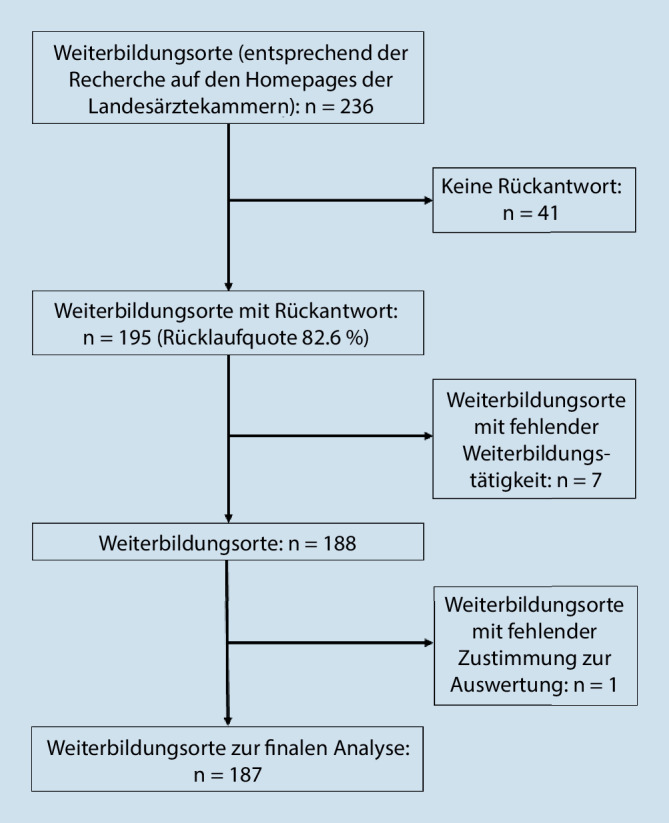

Die Rücklaufquote der zugesandten bzw. online erfassten Fragebögen betrug 82,6 % (195/236); 41 Weiterbildungsorte haben keine Angaben gemacht. Im Rahmen der Befragung konnte festgestellt werden, dass in 7 Einrichtungen keine Weiterbildungsbefugten mehr tätig waren. Es lagen also Angaben von 188 Weiterbildungsstätten vor, in denen zum Zeitpunkt Januar 2021 auch eine Weiterbildung stattfand. Ein Weiterbildungsstandort stimmte der Datenerfassung nicht zu. Somit gehen in die Analyse der Weiterbildungsstellen die Daten von 187 Weiterbildungsorten ein (Abb. 1).

Bezüglich der Weiterbildungsstätten wurde folgende Definition vorgenommen:

*Niederlassung*: Eigenständig geführte Praxis oder Medizinisches Versorgungszentrum (MVZ).

*Kliniken*: Akutkliniken und Rehabilitationskliniken teilweise mit einem ambulanten Versorgungsauftrag.

#### Ethik

Die retrospektive Datenauswertung erfolgte entsprechend den Regularien des Ethikkomitees am Universitätsklinikum Jena.

#### Statistik

Die Daten wurden anhand einer Excel-Tabelle (Microsoft Excel 2016, Redmond, USA) zusammengefasst. Es erfolgte die Durchführung einer deskriptiven Statistik. Die statistischen Analysen wurden mit der Software IBM SPSS Statistics Version 27.0 (IBM SPSS Statistics, Chicago, Illinois, USA) für Windows durchgeführt.

## Ergebnisse

### Weiterbildungsorte

#### Klinik versus Niederlassung

**Figure 2:**
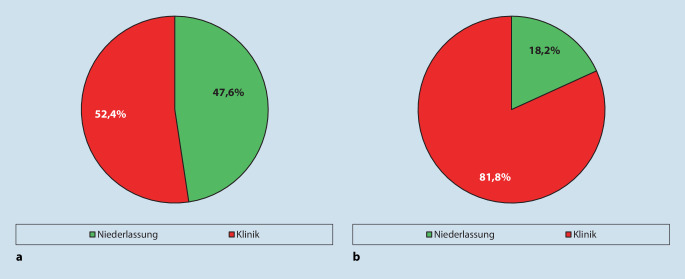

In ganz Deutschland wurden Daten von 187 Weiterbildungseinrichtungen erfasst, welche sich wie folgt auf die Sektoren im Gesundheitssystem aufteilen: Niederlassung (*n* = 89; 47,6 %) und Kliniken (*n* = 98; 52,4 %) (Abb. 2).

Zum Zeitpunkt der Erhebung wurde die Weiterbildung im stationären Bereich v. a. in Universitätskliniken (*n* = 27; 14,4 %) und kommunalen bzw. konfessionellen Kliniken (*n* = 42; 22,5 %) durchgeführt. Privat geführte Kliniken und Rehabilitationskliniken waren an der Weiterbildung mit 9,6 % (*n* = 18) bzw. 5,9 % (*n* = 11) beteiligt. Bezüglich der ambulanten Weiterbildungsorte befanden sich 39,6 % (*n* = 74) in einer eigenständig geführten Praxis und 8,0 % (*n* = 15) in einem medizinischen Versorgungszentrum (MVZ).

#### Weiterbildungsbefugnis und Weiterbildungsdauer

Für 78,6 % (*n* = 147) der Weiterbildungsbefugten lag eine Einzelweiterbildung und bei 21,4 % (*n* = 40) der Weiterbildungsbefugten eine Verbundweiterbildung vor. Die Dauer der Weiterbildungsbefugnis variierte zwischen 6 und 54 Monaten, was sich wie folgt aufgliedert: 18 Monate (38,5 %), 36 Monate (34,8 %) und 12 Monate (17,1 %). In der Niederlassung wurde die Weiterbildung vornehmlich über 18 (71,9 %) bzw. über 12 Monate (19,1 %) durchgeführt. In der Klinik betrug die Weiterbildungszeit meist 36 Monate (62,2 %) (Tab. 1).

**Table 1:** 

Weiterbildungsdauer	Gesamt Anzahl (Prozent)	Niederlassung Anzahl (Prozent)	Klinik Anzahl (Prozent)
Vorläufige Weiterbildungsbefugnis	3 (1,6 %)	2 (2,2 %)	1 (1,0 %)
6 Monate	1 (0,5 %)	1 (1,1 %)	0 (0 %)
12 Monate	32 (17,1 %)	17 (19,1 %)	15 (15,3 %)
18 Monate	72 (38,5 %)	64 (71,9 %)	8 (8,2 %)
24 Monate	5 (2,7 %)	1 (1,1 %)	4 (4,1 %)
30 Monate	4 (2,1 %)	0 (0 %)	4 (4,1 %)
36 Monate	65 (34,8 %)	4 (4,5 %)	61 (62,2 %)
42 Monate	1 (0,5 %)	0 (0 %)	1 (1,0 %)
54 Monate	4 (2,1 %)	0 (0 %)	4 (4,1 %)
*Gesamt*	*187*	*89*	*98*

#### Weiterbildungsorte je Bundesland bzw. Ärztekammer

Die meisten Weiterbildungsstätten befinden sich in Nordrhein-Westfalen (*n* = 38; 20,3 %, mit der Ärztekammer Nordrhein *n* = 23; 12,3 % und der Ärztekammer Westfalen-Lippe *n* = 15; 8,0 %), gefolgt von Bayern (*n* = 29; 15,5 %) und Baden-Württemberg (*n* = 25; 13,3 %). Bremen und das Saarland (*n* = 2; 1,1 %) sowie Schleswig-Holstein und Mecklenburg-Vorpommern (*n* = 5; 2,7 %) weisen die wenigsten Weiterbildungsorte auf (Tab. 2).

**Table 2:** 

	Bundesland (Ärztekammer)	Anzahl der Weiterbildungsstätten (Prozentualer Anteil in Bezug auf die Gesamtweiterbildungsstätten)	Anzahl der Weiterbildungsstellen	Anzahl der besetzten Weiterbildungsstellen (Prozent)	Anzahl der freien Weiterbildungsstellen (Prozent)
1	Baden-Württemberg	25 (13,3 %)	52,0	37,8 (72,7 %)	14,2 (27,3 %)
2	Bayern	29 (15,5 %)	71,0	61,0 (85,9 %)	10,0 (14,1 %)
3	Berlin	12 (6,4 %)	33,7	32,7 (97.0 %)	1,0 (3,0 %)
4	Brandenburg	9 (4,8 %)	16,2	9,2 (56,8 %)	7,0 (43,2 %)
5	Bremen	2 (1,1 %)	4,0	4,0 (100,0 %)	0 (0 %)
6	Hamburg	5 (2,7 %)	11,0	8,5 (77,3 %)	2,5 (22,7 %)
7	Hessen	16 (8,5 %)	37,0	30,5 (82,4 %)	6,5 (17,6 %)
8	Mecklenburg-Vorpommern	5 (2,7 %)	8,0	6,0 (75,0 %)	2,0 (25,0 %)
9	Niedersachsen	11 (5,9 %)	21,0	17,0 (81,0 %)	4,0 (19,0 %)
10	Nordrhein-Westfalen (Ärztekammer Nordrhein)	23 (12,3 %)	46,0	41,0 (89,1 %)	5,0 (10,9 %)
11	Nordrhein-Westfalen (Ärztekammer Westfalen-Lippe)	15 (8,0 %)	72,5	54,0 (74,5 %)	18,5 (25,5 %)
12	Rheinland-Pfalz	3 (1,6 %)	19,5	19,5 (100,0 %)	0 (0 %)
13	Saarland	2 (1,1 %)	4,0	4,0 (100,0 %)	0 (0 %)
14	Sachsen	11 (5,9 %)	24,0	23,0 (95,8 %)	1,0 (4,2 %)
15	Sachsen-Anhalt	8 (4,3 %)	13,5	10,0 (74,1 %)	3,5 (25,9 %)
16	Schleswig-Holstein	5 (2,7 %)	29,0	27,0 (93,1 %)	2,0 (6,9 %)
17	Thüringen	6 (3,2 %)	16,0	11,0 (68,7 %)	5,0 (31,3 %)
–	*Gesamt*	187 (100,0 %)	*478,4*	*396,2 (82,8* *%)*	*82,2 (17,2* *%)*

### Weiterbildungsstellen

#### Weiterbildungsstellen je Bundesland bzw. Ärztekammer

Im Rahmen der in 187 Weiterbildungseinrichtungen durchgeführten Umfrage wurden 478,4 Weiterbildungsstellen für Innere Medizin und Rheumatologie angegeben, von denen 82,8 % (*n* = 396,2) besetzt und 17,2 % (*n* = 82,2) vakant waren. Keine freien Weiterbildungsstellen waren in den Bundesländern Saarland, Rheinland-Pfalz und Bremen zu verzeichnen (Tab. 3).

**Table 3:** 

	Anzahl der Weiterbildungsstellen	Anzahl der besetzten Weiterbildungsstellen (Prozent)	Anzahl der freien Weiterbildungsstellen (Prozent)
Klinik	391,4 (81,8 %)	346,7 (88,6 %)	44,7 (11,4 %)
Niederlassung	87,0 (18,2 %)	49,5 (56,9 %)	37,5 (43,1 %)
*Gesamt*	*478,4*	*396,2 (82,8* *%)*	*82,2 (17,2* *%)*

#### Klinik versus Niederlassung

Die Weiterbildungsstellen zeigten folgende Aufteilung auf die verschiedenen Versorgungsebenen: 81,8 % (*n* = 391,4) in Kliniken und 18,2 % (*n* = 87,0) in der Niederlassung (Tab. 3).

Die Weiterbildungsstellen in Kliniken verteilten sich zu 45,2 % (*n* = 177,0) auf Universitätskliniken, 37,4 % (*n* = 146,4) auf kommunale bzw. konfessionelle Kliniken, 12,8 % (*n* = 50,0) auf private Kliniken und 4,6 % (*n* = 18,0) auf rehabilitative Kliniken (Tab. 4). Bezüglich der Universitätsklinken befanden sich 98 Weiterbildungsstellen an einer eigenständigen rheumatologischen Universitätsklinik, welche durch einen C4- bzw. W3-Lehrstuhl geleitet wird. Für die einem nichtrheumatologischen Lehrstuhl angehörigen rheumatologischen Abteilungen mit einer „nicht weisungsfreien“ W3/W2- oder C3-Professur für Rheumatologie bzw. die Universitäten mit einem rheumatologischen Arbeitsbereich, der einem nichtrheumatologischen Lehrstuhl unterstellt ist, standen weitere 38,5 bzw. 40,5 Weiterbildungsstellen zur Verfügung (Tab. 5).

**Table 4:** 

	Anzahl der Weiterbildungsstellen (Range)	Anzahl der besetzten Weiterbildungsstellen (Prozent)	Anzahl der freien Weiterbildungsstellen (Prozent)
Universitätskliniken	177,0 (1,0–24,0)	162,8 (92,0 %)	14,2 (8,0 %)
Klinik (öffentlicher und konfessioneller Träger)	146,4 (0–17,0)	137,9 (94,2 %)	8,5 (5,8 %)
Klinik (privater Träger)	50,0 (0–7,0)	41,0 (82,0 %)	9,0 (18,0 %)
Rehabilitationsklinik	18,0 (1,0–4,0)	5,0 (27,8 %)	13,0 (72,2 %)
*Gesamt*	*391,4*	*346,7 (88,6* *%)*	*44,7 (11,4* *%)*

**Table 5:** 

Struktur der universitären rheumatologischen Abteilung	Lehrstühle mit Teilnahme an der Umfrage	Weiterbildungsstellen (Prozent)
Eine eigenständige rheumatologische Universitätsklinik durch C4- oder W3-Lehrstuhl für Rheumatologie geleitet	9	98,0 (55,4 %)
Eine einem nichtrheumatologischen Lehrstuhl angehörige rheumatologische Abteilung mit einer „nicht weisungsfreien“ W3/W2- oder C3-Professur für Rheumatologie	8	38,5 (21,8 %)
Universitäten mit einem rheumatologischen Arbeitsbereich, der einem nichtrheumatologischen Lehrstuhl unterstellt ist	10	40,5 (22,8 %)
*Gesamt*	*27*	*177,0*

In der Niederlassung wurden 65,5 % (*n* = 57) der Weiterbildungsstellen in einer eigenständig geführten Praxis und 34,5 % (*n* = 30) der Weiterbildungsstellen in einem medizinischen Versorgungszentrum (MVZ) ermittelt (Tab. 6).

**Table 6:** 

	Anzahl der Weiterbildungsstellen	Anzahl der besetzten Weiterbildungsstellen (Prozent)	Anzahl der freien Weiterbildungsstellen (Prozent)
Eigenständig geführte Praxis	57	27,0 (47,4 %)	30,0 (52,6 %)
MVZ	30,0	22,5 (75,0 %)	7,5 (25,0 %)
*Gesamt*	*87,0*	*49,5 (56,9* *%)*	*37,5 (43,1* *%)*

**Figure 3:**
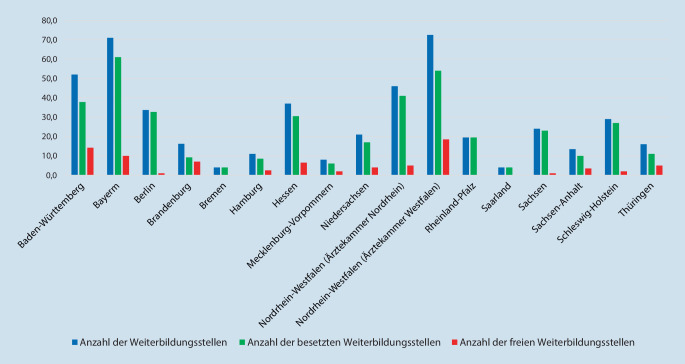

Der Anteil der nicht besetzten Weiterbildungsstellen lag in den Kliniken bei 11,4 % (8,0 % in Universitätskliniken, bei 5,8 % in öffentlichen und konfessionellen Kliniken, bei 18,0 % in privaten Kliniken und bei 72,2 % in Rehabilitationskliniken) und im ambulanten Bereich bei 43,1 % der Weiterbildungsstellen (52,6 % in einer eigenständig geführten Praxis bzw. 25 % in einem MVZ) (Abb. 3, 4 und 5).

**Figure 4:**
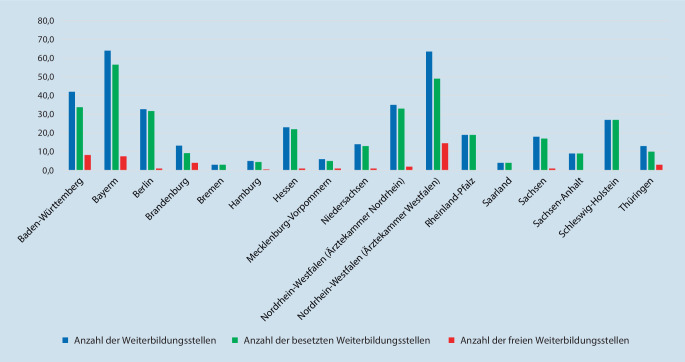

**Figure 5:**
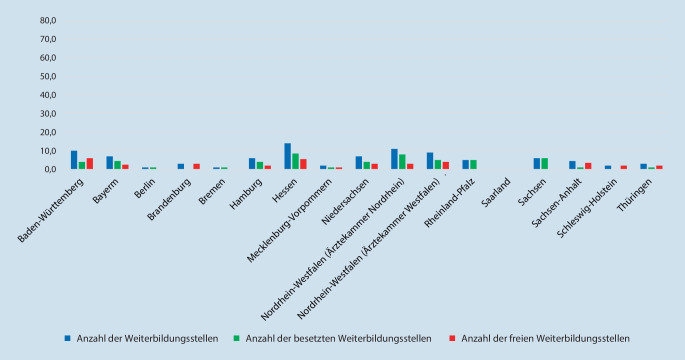

Insgesamt waren 92 Weiterbildungsstellen durch Teilzeitkräfte besetzt (Tab. 7).

**Table 7:** 

		Anzahl
*Teilzeitstellen*	80 %	21
	75 %	26
	60 %	15
	50 %	29
	40 %	1
	*Gesamt*	*92*

## Diskussion

Basierend auf den Forderungen des „Bündnisses für Rheumatologie“ zur Sicherstellung der rheumatologischen Versorgung und der Bedarfsplanung der DGRh unter Berücksichtigung der fachinternistischen Planung der Kassenärztlichen Bundesvereinigung werden über 1000 Fachärztinnen und Fachärzte für Innere Medizin und Rheumatologie für eine bedarfsgerechte rheumatologische Versorgung benötigt [5]. Eine Aufrechterhaltung und mögliche Verbesserung der Versorgungssituation ist nur durch die Aus- und Weiterbildung neuer Fachärztinnen und Fachärzte für Innere Medizin und Rheumatologie möglich. Aus diesem Grund hat der Vorstand der DGRh seiner Kommission Weiterbildung die hier vorgelegte Studie zur Erfassung der Weiterbildungsorte und Weiterbildungsstellen für Innere Medizin und Rheumatologie in Deutschland in Auftrag gegeben.

### Weiterbildungsorte

Durch die Online-Recherche der Webseiten der Landesärztekammern wurden 236 Weiterbildungsorte für die Weiterbildung zur Fachärztin bzw. zum Facharzt für Innere Medizin und Rheumatologie eruiert, wobei für 187 Weiterbildungsorte eine vollständige Umfrage vorlag. In der Publikation von Braun et al. aus dem Jahr 2011 wurde noch berichtet, dass bei 155 Weiterbildungsbefugten eine rheumatologische Weiterbildung stattfindet [2]. Das könnte bedeuten, dass es in den letzten 10 Jahren eine tendenzielle Zunahme der Weiterbildungsorte gegeben hat.

Die Weiterbildungsorte verteilten sich im Jahr 2021 mit 47,6 % auf Niederlassung und mit 52,4 % auf Klinik (insbesondere kommunale bzw. konfessionell geführte Kliniken und Universitätskliniken). Im ambulanten Bereich waren die Weiterbildungsstellen (83,1 %) hauptsächlich in Einzelpraxen lokalisiert. Also war knapp die Hälfte der Weiterbildungsorte im ambulanten Bereich und etwas mehr als die Hälfte in Kliniken angesiedelt. Allerdings waren die meisten Stellen nicht besetzt.

Die meisten Weiterbildungsorte befinden sich in Nordrhein-Westfalen (20,3 %), Bayern (15,5 %) und Baden-Württemberg (13,4 %). Hiermit zeigen die bevölkerungsstarken Bundesländer eine deutlich höhere Dichte an Weiterbildungsorten im Vergleich zu den Bundesländern mit einer niedrigeren Einwohnerzahl.

### Weiterbildungsstellen

Insgesamt konnten in Deutschland 478,4 Weiterbildungsstellen für Innere Medizin und Rheumatologie durch die Umfrage erfasst werden. Zum Erhebungszeitpunkt waren 82,8 % der Weiterbildungsstellen besetzt, und 17,2 % der Weiterbildungsstellen waren nicht besetzt. Die meisten Weiterbildungsstellen waren in der Klinik (81,8 %) vorhanden. Gegenüber den Weiterbildungsorten, die zu knapp der Hälfte im niedergelassenen Sektor lokalisiert sind, werden nur 18,2 % der Weiterbildungsstellen dem ambulanten Sektor zugeordnet.

Die meisten Weiterbildungsstellen sind an Universitätskliniken (*n* = 177,0) und kommunalen bzw. konfessionellen Kliniken (*n* = 146,4) angesiedelt. Diese Daten stimmen mit der Umfrage der Arbeitsgemeinschaft Junger Rheumatologen in der DGRh von 2018 überein, welche ebenfalls die größte Anzahl von Ausbildungsstellen an Universitätskliniken (*n* = 49) ermittelt hatte. Damals schien nur ein sehr geringer Teil der Ausbildung im ambulanten Bereich (*n* = 1) durchgeführt zu werden [9]. Unter Berücksichtigung der Universitätskliniken als Hauptausbildungsstätte für Rheumatolog:innen konnte in den letzten Jahren eine leichte Zunahme der universitären rheumatologischen Abteilungen verzeichnet werden. In der Rhesus-Studie (Rheumatologische Strukturen an universitären Standorten) aus dem Jahr 2008 wurden noch 6 eigenständige rheumatologische Universitätskliniken und 5 eigenständige rheumatologische Abteilungen als Funktionsbereich einer größeren Klinik ermittelt [8]. In der 2016 publizierten RISA-Studie III (Rheumatologie – Integration in die studentische Ausbildung) konnten 7 eigenständige rheumatologische Universitätskliniken und 9 rheumatologisch geführte Abteilungen unter einem nichtrheumatologischen Lehrstuhl quantifiziert werden [14]. Aktuell sind in Deutschland 9 eigenständige rheumatologische Universitätskliniken (Berlin [Charité Campus Mitte], Bielefeld/Oldenburg, Bochum/Herne, Düsseldorf, Erlangen, Freiburg, Gießen, Hannover und Lübeck) sowie 11 rheumatologisch geführte Abteilungen unter einem nichtrheumatologischen Lehrstuhl (Berlin [Campus Benjamin Franklin], Dresden, Frankfurt/Main, Hamburg, Heidelberg, Kiel, Leipzig, Mainz, München [Ludwig-Maximilians-Universität], Münster und Regensburg/Bad Abbach) vorhanden. Anhand der Daten kann gezeigt werden, dass es in den letzten Jahren einen leichten Zuwachs der rheumatologischen Lehrstühle an den Universitätskliniken gegeben hat. Es ist zu hoffen, dass dies möglicherweise zu einer besseren Stellensituation in der universitären rheumatologischen Weiterbildung geführt hat.

Zum Erhebungszeitpunkt waren 17,2 % der Weiterbildungsstellen nicht besetzt. In der Publikation von Braun et al. wurde berichtet, dass 2011 nur 65 % der Weiterbildungsermächtigten eine Weiterbildung zur Fachärztin bzw. Facharzt für Rheumatologie durchführen [2]. Hieraus resultiert, dass bei 35 % der Weiterbildungsermächtigten keine Weiterbildung durchgeführt wird. Ob das jetzt noch so ist, ist unklar, aber möglicherweise bedeutet dies eine Abnahme der freien Weiterbildungsstellen.

In diesem Zusammenhang ist anhand der Daten der aktuellen Studie festzustellen, dass 43,1 % der Weiterbildungsstellen im ambulanten Bereich nicht besetzt sind. Als eine mögliche Ursache ist die fehlende Finanzierung der Weiterbildungsstellen durch die Kassenärztlichen Vereinigungen (KV) zu berücksichtigen, welche von politischer Seite gefordert und vorangetrieben werden müsste. Des Weiteren wird von den meisten KVen eine Zunahme der Patientenzahlen bei der Beschäftigung von Weiterbildungsassistent:innen abgelehnt. Grundsätzlich ist die Politik gefordert, die Sicherstellung der rheumatologischen Versorgung durch eine adäquate finanzielle Unterstützung der Weiterbildung im ambulanten und stationären Bereich zu gewährleisten.

### Ausblick

Für die Zukunft des Fachgebietes Innere Medizin und Rheumatologie ist es von besonderer Bedeutung, die bestehenden ambulanten und klinischen Ausbildungskapazitäten zu erhalten und optimal zu nutzen [3]. Hierfür müssen sektorübergreifende Weiterbildungskonzepte entwickelt und eine eigenständige Vergütung des Weiterbildungsaufwandes etabliert werden.

Sollte eine Finanzierung der Weiterbildungsstellen durch die KV möglich sein (in Analogie zum Fachgebiet Allgemeinmedizin), könnte das Potenzial ambulanter Weiterbildungsstellen zur Aus- und Weiterbildung neuer Rheumatolog:innen besser ausgeschöpft werden, zumal die Hälfte der Weiterbildungsorte im ambulanten Bereich de facto vorhanden ist. In diesem Zusammenhang sollte die kassenärztliche Bundesvereinigung, die Deutsche Krankenhausgesellschaft und der (GKV-)Spitzenverband der gesetzlichen Krankenversicherung gemäß dem § 75a SGB V die Verpflichtung zur allgemeinärztlichen Weiterbildung in Vertragsarztpraxen und MVZ auf das Fachgebiet Rheumatologie ausweiten [5]. Aus diesem Grund fordert der Berufsverband der deutschen Rheumatolog:innen (BdRh) eine Förderung der ambulanten Weiterbildung [3], um die Versorgungslücke zu verringern [5].

Eine Vergütung des Weiterbildungsaufwandes ist auch im fallpauschalisierten Entgeltsystem (DRG-System) nicht abgebildet, weshalb auch für den klinischen Sektor ein adäquater Ausgleich für Weiterbildungsstellen zu fordern ist. Die Fallpauschalen führen zu einer Stärkung der umsatz- und gewinnstarken Abteilungen mit einer entsprechenden Ausstattung an Weiterbildungsstellen entsprechend dem Budget bzw. Gewinn einer Fachabteilung [5]. Folglich muss eine Entkopplung der Krankenhausfinanzierung und der Finanzierung der Weiterbildungsstellen erfolgen [10]. In diesem Zusammenhang verweist das Bündnis Rheumatologie auf andere europäische Länder, bei denen die Zahl der Weiterbildungsstellen steuerlich finanziert und an dem Versorgungsbedarf der Bevölkerung festgelegt wird mit der Folge eines fehlenden rheumatologischen Facharztmangels [10]. Des Weiteren ist einer weiteren Reduktion der stationären rheumatologischen Versorgungskapazität dringend entgegenzuwirken. Nur mit dem Erhalt der rheumatologischen Akutkliniken wird die Weiterbildung nachhaltig gesichert [10].

Die Einbeziehung der ambulanten Rheumatologie in sektorübergreifende Weiterbildungskonzepte durch eine strukturierte Zusammenarbeit mit standortnahen klinischen Weiterbildungsstätten könnte ebenfalls die Attraktivität einer rheumatologischen Weiterbildung erhöhen und zu einer Verbesserung der Versorgungssituation führen.

Der Hauptteil der Weiterbildungsstellen ist im universitären Sektor angesiedelt. Innerhalb der Universitätskliniken sind die meisten Stellen an eigenständigen rheumatologischen Abteilungen mit einer eigenständigen Leitung durch eine C4- bzw. W3-Professur angesiedelt. An den 38 Fakultäten in Deutschland sind 9 eigenständige rheumatologische Abteilungen und 11 nicht weisungsfreie Professorinnen und Professoren mit eigener rheumatologischer Abteilung vorhanden. In diesem Zusammenhang ist ein weiterer Ausbau von eigenständigen rheumatologischen Abteilungen an den Universitätskliniken zu fordern, um die studentische Ausbildung zu verbessern und mehr universitäre Weiterbildungsstellen zu schaffen.

An dieser Stelle ist auch anzumerken, dass mit der Einführung der Musterweiterbildungsordnung 2018 und durch die Implementierung des Mustercurriculums der DGRh zur Weiterbildung im Fachgebiet Innere Medizin und Rheumatologie die Basis für eine standardisierte Weiterbildung im ambulanten und stationären Sektor geschaffen worden ist [4, 13].

## Schlussfolgerung

In Deutschland befindet sich jeweils etwa die Hälfte der Weiterbildungsstätten zur Erlangung der Weiterbildungsbezeichnung Fachärztin bzw. Facharzt für Rheumatologie in den Kliniken und im niedergelassenen Bereich. Die weit überwiegende Mehrzahl der Weiterbildungsstellen ist allerdings im klinischen Sektor vorhanden. Zudem ist die Hälfte der Weiterbildungsstellen im ambulanten Bereich nicht besetzt. Aus diesem Grund sollten für eine optimale Nutzung bereits bestehender Weiterbildungskapazitäten sektorübergreifende Weiterbildungskonzepte entwickelt und eine eigenständige Vergütung des Weiterbildungsaufwandes etabliert werden. Hierzu zählt die gesetzlich verpflichtende Förderung der ambulanten Weiterbildung (§ 75a SGB V) und die Bemessung der stationären Weiterbildungsstellen entsprechend dem prognostiziertem Versorgungsbedarf der Bevölkerung wie vom „Bündnis für Rheumatologie“ gefordert [5, 10]. Nur auf diesem Wege ist eine adäquate rheumatologische Versorgung der Bevölkerung zu realisieren.

## Fazit für die Praxis

- Aktuell findet die Weiterbildung zur Fachärztin bzw. zum Facharzt für Innere Medizin und Rheumatologie hauptsächlich im klinischen Bereich statt.
- Die Niederlassung sollte weit mehr in die Weiterbildung eingebunden werden.
- Für eine optimale Nutzung bereits bestehender Weiterbildungskapazitäten sollten sektorübergreifende Weiterbildungskonzepte entwickelt und eine eigenständige Vergütung des Weiterbildungsaufwandes etabliert werden.
- Definition der Anzahl der Weiterbildungsstellen am Versorgungsbedarf der Bevölkerung.

## Abkürzungen

DGRh: Deutsche Gesellschaft für Rheumatologie
MVZ: Medizinisches Versorgungszentrum

## References

[1] Al Maini M, Adelowo F, Al Saleh J, Al Weshahi Y, Burmester GR, Cutolo M, Flood J, March L, McDonald-Blumer H, Pile K, Pineda C, Thorne C, Kvien TK (2015). The global challenges and opportunities in the practice of rheumatology: white paper by the World Forum on Rheumatic and Musculoskeletal Diseases. Clin Rheumatol.

[2] Braun J, Wollenhaupt J, Genth E (2011). Further education: a core responsibility of the German Society for Rheumatology and the Rheuma Academy. Z Rheumatol.

[3] Fiehn C, Baraliakos X, Edelmann E, Froschauer S, Feist E, Karberg K, Ruehlmann JM, Schuch F, Welcker M, Zinke S (2020). Current state, goals and quality standards of outpatient care in rheumatology: position paper of the Professional Association of German Rheumatologists (BDRh). Z Rheumatol.

[4] Fleck M, Berliner MN, Krause A (2021). Novel revision of the training regulations for German physicians: consequences for trainees and trainers in rheumatology. Z Rheumatol.

[5] (2021) Forderung des „Bündnisses für Rheumatologie“ zur Sicherstellung der rheumatologischen Versorgung. https://dgrh.de/dam/jcr:7bbd1ea7-6637-4d2b-ab38-9e00d4a641df/BFRh_9_Statement_Forderungen_211101.pdf. Zugegriffen: 14.10.2022

[6] (2020) https://www.bundesaerztekammer.de/fileadmin/user_upload/_old-files/downloads/pdf-Ordner/Statistik_2020/Tabelle_9-Anerkennung_von_Facharztbezeichnungen.pdf. Zugegriffen: 14.10.2022

[7] Keysser G, Baerwald CGO, Sieburg M, Boche K, Pfeil A, Kupka TA, Luthke K, Heldmann F, Oelzner P, Unger L, Aringer M (2019). Survey of rheumatologists in Saxony, Saxony-Anhalt and Thuringia regarding the occupational situation and activities in further education: no way out of the undersupply of rheumatological care. Z Rheumatol.

[8] Keysser G, Burmester GR (2008). The current structure of rheumatology in German universities. Z Rheumatol.

[9] Krusche M, Sewerin P, Kleyer A, Mucke J, Vossen D, Morf H (2019). Specialist training quo vadis?. Z Rheumatol.

[10] Lorenz HM, Froschauer S, Hanke R, Hellmich B, Krause A, Lakomek HJ, Kötter I, Strunk J, Voormann A, Zinke S (2022). Publicity campaign rheuma2025 of the Union for Rheumatology. Z Rheumatol.

[11] Pfeil A, Baerwald CGO, Sieburg M, Boche K, Kupka TA, Linde T, Heldmann F, Unger L, Oelzner P, Aringer M, Keysser G (2020). Future of rheumatologists: what are the perspectives? Survey of resident physicians in rheumatology in middle Germany. Z Rheumatol.

[12] Pfeil A, Fleck M, Keyßer G (2021). Specialist training situation in rheumatology from a trainer’s perspective. Z Rheumatol.

[13] Pfeil A, Krusche M, Vossen D, Berliner MN, Keyßer G, Krause A, Lorenz HM, Manger B, Schuch F, Specker C, Wollenhaupt J, Baraliakos X, Fleck M, Proft F (2021). Model curriculum of the German Society for Rheumatology for advanced training in the discipline internal medicine and rheumatology. Z Rheumatol.

[14] Riemekasten G, Aringer M, Baerwald CG, Meyer-Bahlburg A, Bergner R, Feuchtenberger M, Gebhardt C, Hellmich B, Keysser G, Lorenz HM, Kneitz C, Witte T, Muller-Ladner U, Schneider M, Braun J, Rautenstrauch J, Specker C, Schulze-Koops H (2016). Rheumatology—Integration into student training (RISA): current structure of clinical rheumatology in German universities (RISA III). Z Rheumatol.

[15] Smith E, Hoy DG, Cross M, Vos T, Naghavi M, Buchbinder R, Woolf AD, March L (2014). The global burden of other musculoskeletal disorders: estimates from the Global Burden of Disease 2010 study. Ann Rheum Dis.

[16] Zink A, Braun J, Gromnica-Ihle E, Krause D, Lakomek HJ, Mau W, Muller-Ladner U, Rautenstrauch J, Specker C, Schneider M (2017). Memorandum of the German Society for Rheumatology on the quality of treatment in rheumatology—Update 2016. Z Rheumatol.

